# Development of 3D Hepatic Constructs Within Polysaccharide-Based Scaffolds with Tunable Properties

**DOI:** 10.3390/ijms21103644

**Published:** 2020-05-21

**Authors:** Marie-Noëlle Labour, Camile Le Guilcher, Rachida Aid-Launais, Nour El Samad, Soraya Lanouar, Teresa Simon-Yarza, Didier Letourneur

**Affiliations:** 1 INSERM U1148, LVTS, Université de Paris, X Bichat Hospital, 46 rue H Huchard, F-75018 Paris, France; marie-noelle.labour@enscm.fr (M.-N.L.); camille.le-guilcher@inserm.fr (C.L.G.); rachida.aid@inserm.fr (R.A.-L.); nourelsamad@hotmail.com (N.E.S.); soraya.lanouar@gmail.com (S.L.); teresa.simon-yarza@inserm.fr (T.S.-Y.); 2 INSERM U1148, LVTS, Université Sorbonne Paris Nord, 99 av JB Clément, 93430 Villetaneuse, France; 3 École Pratique des Hautes Études, Paris Sciences et Lettres (PSL) Research University, 4-14 rue Ferrus, 75014 Paris, France; 4 INSERM UMS-34, FRIM Université de Paris, X Bichat School of Medicine, F-75018 Paris, France

**Keywords:** 3D scaffold, tissue engineering, polysaccharide, organoid, liver, HepG2

## Abstract

Organoids production is a key tool for in vitro studies of physiopathological conditions, drug-induced toxicity assays, and for a potential use in regenerative medicine. Hence, it prompted studies on hepatic organoids and liver regeneration. Numerous attempts to produce hepatic constructs had often limited success due to a lack of viability or functionality. Moreover, most products could not be translated for clinical studies. The aim of this study was to develop functional and viable hepatic constructs using a 3D porous scaffold with an adjustable structure, devoid of any animal component, that could also be used as an in vivo implantable system. We used a combination of pharmaceutical grade pullulan and dextran with different porogen formulations to form crosslinked scaffolds with macroporosity ranging from 30 µm to several hundreds of microns. Polysaccharide scaffolds were easy to prepare and to handle, and allowed confocal observations thanks to their transparency. A simple seeding method allowed a rapid impregnation of the scaffolds with HepG2 cells and a homogeneous cell distribution within the scaffolds. Cells were viable over seven days and form spheroids of various geometries and sizes. Cells in 3D express hepatic markers albumin, HNF4α and CYP3A4, start to polarize and were sensitive to acetaminophen in a concentration-dependant manner. Therefore, this study depicts a proof of concept for organoid production in 3D scaffolds that could be prepared under GMP conditions for reliable drug-induced toxicity studies and for liver tissue engineering.

## 1. Introduction

The liver is a vital organ with many crucial functions of detoxification, lipid and carbohydrate metabolism, homeostasis maintenance or blood protein synthesis. These functions can be impaired in many pathological conditions such hepatitis, cancer, genetic diseases or long term medication that induce liver fibrosis followed by cirrhosis which is irreversible and often leads to liver failure associated with poor prognosis. Cirrhosis and hepatocellular carcinoma are accountable for about 3.5% of deaths worldwide with an increasing prevalence [1]. Currently, allografts are the gold standard to treat end-stage liver diseases but, due to the lack of donors, tissue engineering strategies represent a promising therapeutic route for liver diseases [2,3,4,5]. Moreover, with the aim to reduce drug toxicity testing on animal models, tissue engineering platforms can be used as an efficient tool to reproduce liver architecture. Indeed, three-dimensional (3D) models have been extensively developed as an alternative to two-dimensional (2D) cell culture models that do not mimic complex tissues structures, and the benefits of 3D culture have been broadly demonstrated for a variety of cell types. Organoid biotechnologies emerged from the idea to recapitulate organogenesis and morphogenesis in order to create in vitro “organ on a chip” systems [6,7]. This technology is based on cell self-assembly, differentiation and production of extracellular matrix to produce a system mimicking organ structures. This strategy represents a highly valuable tool in particular for disease modeling. As hepatocytes rapidly lose their phenotype and specific functions when cultured in 2D [8,9,10], this approach has been largely investigated to improve in vitro models for drug-toxicity and liver diseases [11] or for tissue engineering applications including extracorporeal [11,12,13,14] or implantable devices to sustain liver functions during acute phases of diseases [5,15].

In the last decade, a variety of techniques have been developed or improved to produce functional hepatic organoids from self-assembly in microwells and microfluidic devices to hydrogels and additive manufacturing technologies [9,16,17,18,19,20]. Initially, sandwiched cultures promoted survival of functional primary hepatocytes and have been widely used since then [21,22]. Later on, spheroids were produced using different simple techniques such as culture on non-adherent plates, stirred plates or bioreactors [23,24,25,26], that offered spheroid mass production. To improve reproducibility and produce organoids with a homogeneous size, hanging-drop and emulsion-based techniques [19], as well as microwells made of non-adherent materials, were developed [18,27,28,29]. Some of these systems have led to product commercialization, for instance microwells Gri3D™ that can be used for different applications or preformed liver organoids 3D InSight™ Liver Microtissues. Importantly, hepatic organoid technology combined with microfluidic devices enabled high throughput screening for drug toxicity studies and improved in vitro models of liver fibrosis or tumor microenvironments [19,30,31]. Besides, Takebe et al. successfully implanted vascularized liver buds in mice of that sustain major liver functions [4]. However, drawbacks of these systems are the large diameter of the microtissues generated that could induce cell necrosis, the lack of functionality and most often the difficulty to manipulate these systems. To overcome these limitations, 3D scaffolds have been considered for hepatic organoid production. These scaffolds are frequently hydrogels made of natural materials as they exhibit soft mechanical properties similar to healthy liver [7,16,32,33,34]. The main constraint of 3D matrices for organoid studies are porosity and pore interconnectivity to allow cell infiltration and generation of organoids but also nutrients diffusion [9,19,26]. Matrigel, a protein mixture secreted by Engelbreth-Holm-Swarm mouse sarcoma cells, has been extensively used as support material for 3D cell culture, but it exhibits a low porosity and a high variability in terms of cell response, and cannot be clinically approved [10,35,36,37]. To favor cell–cell contacts and hence organoid formation, materials that do not present cell adhesion motifs are often used, in particular alginate-based matrices that promote hepatocyte spheroid formation by enhancing cell–cell interactions as well as hepatocellular activity such as albumin and urea production or detoxification functions [33,36,38]. All these systems are promising for the production of functional organoids but there is still a need to produce 3D scaffolds, easy to manipulate with a process that can be translated to the clinic with industrial processes. In addition, the 3D platforms should allow an easy loading of a high number of cells, have open and interconnected pores within the 3D structure for cell self-assembly, cell–cell contacts and matrix production while maintaining high cell viability.

The aim of our study was to produce 3D porous scaffolds with adjustable properties for viable and functional organoid development, allowing easy handling and observations. The challenges were to obtain highly porous structures that allow efficient cell loading and self-assembly into viable and functional organoids. In this context, we took the benefit of pullulan-dextran 3D porous matrices already used for in vitro and in vivo tissue engineering studies [39,40,41,42]. The main advantages of pullulan and dextran, two natural water-soluble polysaccharides already used in the food and pharmaceutical industry, are their biocompatibility and biodegradability. While dextran provides stability thanks to its high molecular weight, pullulan provides flexibility to the scaffold thanks to its branched polymeric structure. This combination produced a scaffold with soft and elastic mechanical properties easy to manipulate. These scaffolds have been used for a wide variety of applications such as vascular or bone tissue engineering [39,43,44,45] and their biodegradability is dependent on crosslinking ratio [46]. Moreover, they can be produced under GMP-conditions at large-scale and have been successfully implanted without major inflammatory reaction in mice, rats and goats [39,43,47]. The process for hydrogel formation relies on the addition of porogens during crosslinking using sodium trimetaphosphate (STMP) and freeze drying, a simple method to produce scaffolds easily at low cost and without any organic solvent. In this study, we describe for the first time the self-assembly of hepatic constructs in pullulan-dextran porous matrices. By varying porogen type and amounts, we have prepared several types of matrices and used HepG2 cell line as a model to investigate the effect of the formulation, and more particularly, the effect of porosity on cell clusters size and shape, cell viability and liver-specific functions.

## 2. Results

### 2.1. Scaffold Preparation and Characterization

The first objective of this study was to produce scaffolds to study the effect of different porosities on the viability and functionality of hepatic constructs. We used a patented method to create pullulan-dextran hydrogels based on the addition of porogens and a freeze-drying process [41,42]. A ratio 75:25 of high molecular weight Pullulan:Dextran has already been optimized to obtain easy-to-handle scaffolds [39,40,41,42]. In the present study, we investigated the effects of different amounts of two porogens, namely sodium chloride (NaCl) and sodium carbonate (Na_2_CO_3_) to obtain porous scaffolds that maximize cell loading and the diffusion of nutrients, oxygen and waste products. The limiting factor to increase porogen concentration is that polysaccharide solutions are very viscous. In the case of NaCl, a maximum of 17% (*w/w*) of porogen could be added in the polysaccharide solution (Table 1). For Na_2_CO_3_, a maximum was reached at 15% w/w. At 18% Na_2_CO_3_, although the solution appeared homogeneous, the procedure for scaffold molding was hindered by the too high viscosity (Table 1), and these scaffolds could not be obtained. Combined formulations with both NaCl and Na_2_CO_3_ were also prepared. With the combined formulations (ratio NaCl:Na_2_CO_3_ 1:1), scaffolds with a total weight of 15% and 20% (*w/w*) could be obtained, and were named Combined-15 and Combined-20, respectively.

Physicochemical properties of the scaffolds were then analyzed. We studied phosphorous content that appraises crosslinking degree, swelling ratio and enzymatic degradation. Phosphorous content analysis revealed a significantly higher phosphorous content in NaCl scaffolds than in all other scaffolds suggesting a higher crosslinking ratio (Table 1). Swelling ratio was different for the five formulations, with Combined-20 scaffold being the highest. This observation implies that swelling does not depend primarily on phosphorous content since the latter was minimal for Na_2_CO_3_-15, maximal for NaCl, and an intermediate value for Combined-20 scaffolds. Enzymatic degradation analysis revealed that scaffold degradation time is the longest in NaCl scaffolds which present the highest phosphorous content. For other formulations, where phosphorous content is similar, degradation t_1/2_ decreases with an increasing amount of porogen (Table 1).

**Figure 1:**
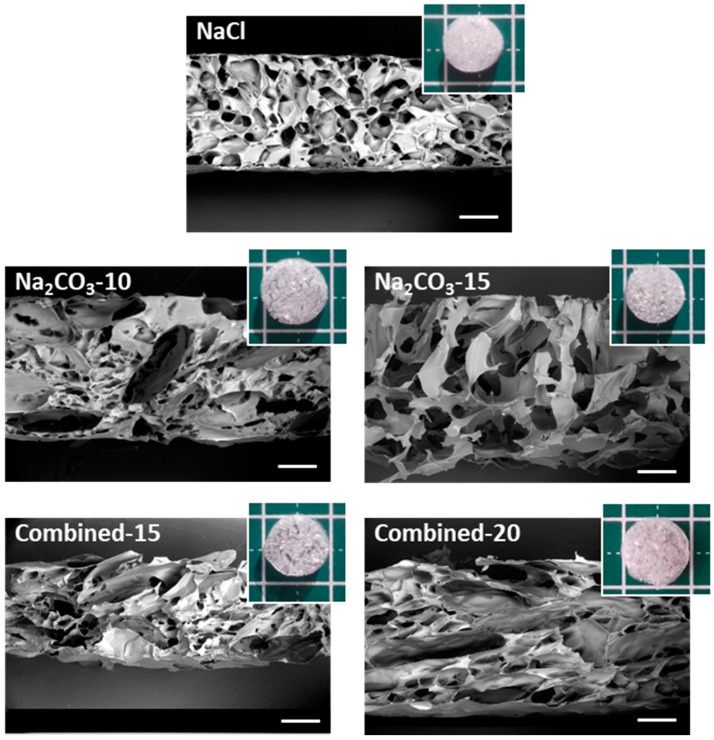
Macroscopic and microscopic (SEM) analysis of scaffold structures in dry state. Scaffolds for the five selected formulations were cut vertically to observe by SEM the inner structure under low vacuum mode (40 Pa) at a 20-kV acceleration voltage. Scale bars on SEM pictures are 500 µm. Five representative dry scaffolds placed on a 1 × 1 cm mat are shown in inserts.

The macro- and micro-structures of the scaffolds after freeze-drying were analyzed by SEM to observe the internal porosity in dry state. Macroscopically, the structure of scaffolds formed with NaCl appears dense compared to all other formulations, where pores were even visible with the naked eye (Figure 1). The Combined-20 scaffold presents a distinctive cotton-like structure. SEM observations demonstrate that all dry scaffolds possess an open structure with interconnected pores. NaCl scaffolds present a homogeneous pore size distribution of approximately 200 μm and a circular shape. In contrast, Na_2_CO_3_-10 scaffolds present two types of porosity: (i) small pores similar in shape to the ones obtained with NaCl alone and (ii) more elongated pores (width ≈ 200 μm, length ≈ 1 mm), likely created by CO_2_ release in the washing acidic step. In Na_2_CO_3_-15 scaffolds, we mainly observed very large pores of about 500 µm with irregular sizes. Thus, a mix of large elongated and small pores was obtained for combined scaffolds with the sizes increasing as the porogen concentration increases (Figure 1).

**Figure 2:**
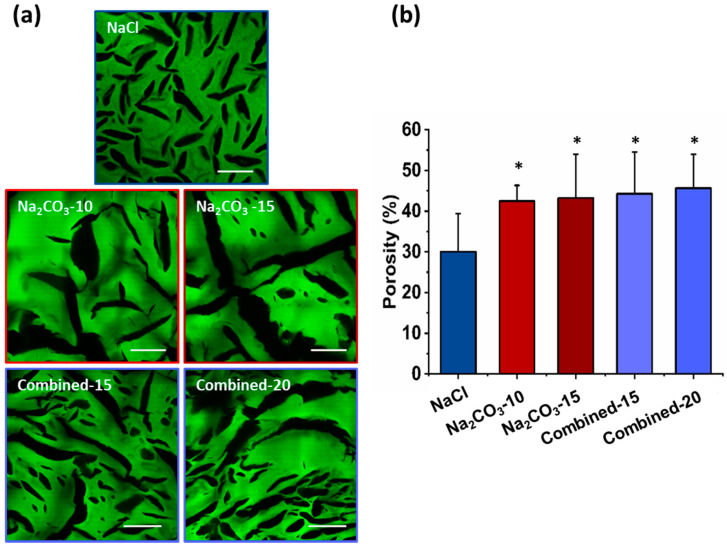
Hydrogels’ porosity after hydration. (**a**) Representative confocal microscopy images of the different scaffolds containing Dextran-FITC. Scale bar 200µm. (**b**) Scaffolds’ porosity calculated from confocal stacks images in A. Statistics were performed using student *t*-test. Only NaCl scaffold porosity was significantly different than every other one. * *p* < 0.05, *n* = 6.

The microscopic structures of hydrated scaffolds were analyzed using 1% FITC-dextran included in the initial formulations. Upon scaffold hydration in PBS, polysaccharide scaffolds immediately retracted due to hygroscopic properties of polysaccharides and then swelled within less than a minute, and produced transparent hydrogels. All scaffolds were easy to handle, soft and cohesive. Upon hydration, all pore sizes appear slightly smaller than the dry scaffolds but present a similar shape (Figure 2a). Scaffolds prepared with NaCl exhibited ovoid pores of about 200 µm in length and about 30 µm in width, whereas scaffolds with Na_2_CO_3_ presented more elongated pores between 0.5–1 mm length and widths ranging from 30 and 150 µm. In hydrated Na_2_CO_3_ scaffolds, we did not notice the smaller pores as observed in NaCl scaffolds. Regarding combined scaffolds, both types of porosity were observed: (i) small and ovoid; (ii) thin and elongated pores. Elongated pores in combined scaffolds were less present than in Na_2_CO_3_ scaffolds and had widths (30–60 µm) lower than in Na_2_CO_3_ scaffolds. Smaller pores appeared similar to NaCl scaffolds although they presented a larger distribution in terms of size and geometry. No obvious difference was noted when increasing porogen concentrations. The percentage of porosity in volume was analyzed quantitatively in 3D stacks. For NaCl scaffolds, total porosity was about 30% whereas for all other conditions, porosity was about 40–50% (Figure 2b). All porous scaffolds presented a high interconnectivity of pores within the entire 3D structures.

### 2.2. Cell Cluster Size and Geometry

**Figure 3:**
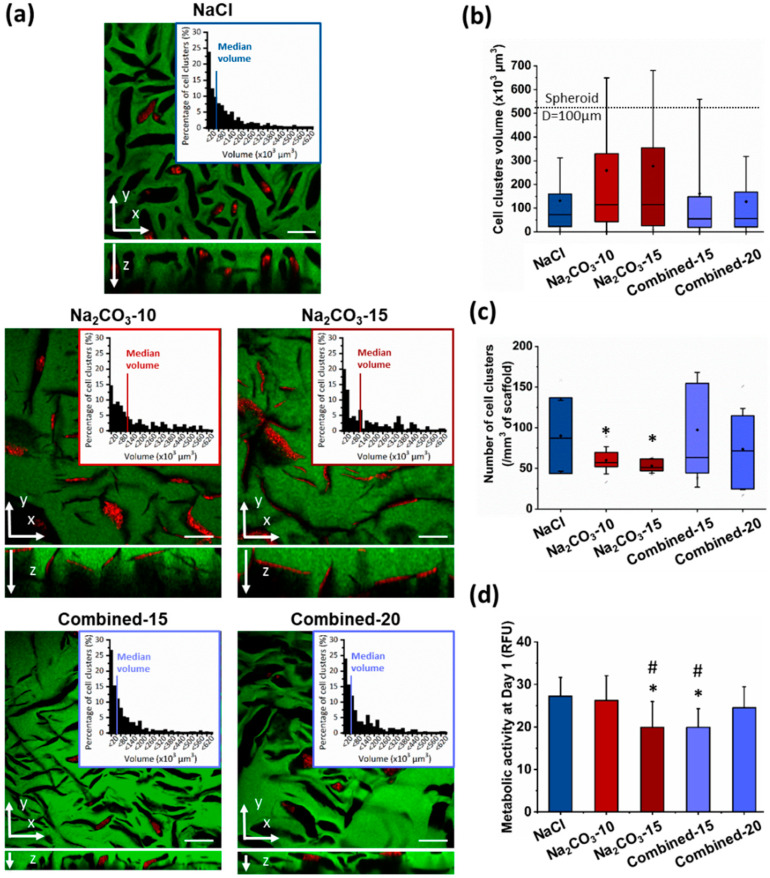
HepG2 cell clusters size and conformation in 3D scaffolds after seven days of culture. (**a**) Confocal image of the interior of scaffolds formed using NaCl (top panel), Na_2_CO_3_ (middle panel) or a combination of both porogens (bottom panel). Scaffolds were observed here in green due to 1% Dextran-FITC in the formulations. Cell clusters are shown using phalloidin-TRITC staining. Volume distribution of cell clusters (top right panels) formed within the scaffolds were determined using a 3D semi-automatic analysis using Imaris software. Scale bar: 200µm. (**b**,**c**) Distribution of cell clusters volumes (**b**) and number of cell clusters per mm^3^ of scaffold (**c**) represented as box plot using median and 25–75 percentile. Mean volume is represented as a diamond-shape dot, error bars represent standard deviation. (**d**) Metabolic activity analysed at day one after seeding. Statistical analysis was performed using student *t*-test. * represent statistical difference towards NaCl scaffolds. ^#^ represent statistical difference (*p* < 0.05) towards Na_2_CO_3_-10 scaffolds. All other differences were not significant.

Next, we investigated whether a change of porosity would modify geometry and size of cell clusters, and hence influence cell self-assembly, viability and functionality [17,26,29]. Indeed, to form viable and functional organoids in a 3D scaffold, one main factor is cell cluster size to allow nutrients and oxygen diffusion to the cells in the center of the construct, a critical feature for hepatic cells that requires high concentrations in glucose. Therefore, HepG2 cells were observed in the different scaffolds after seven days, stained for phalloidin and DAPI and imaged by confocal microscopy to observe cell clusters. We could see that cells were successfully loaded in all the scaffolds with a homogeneous distribution throughout the entire hydrogel. In NaCl scaffolds, where pores were small and ovoid, cell clusters appear small and circular, and they did not colonize the entire volume of the pores (Figure 3a top panel). Size analysis revealed a majority of small cell clusters with a median volume of 72.1 µm^3^, i.e., about 52 µm in diameter if considered as spherical (Figure 3a,b). In NaCl scaffolds, only 4.4% of cell clusters presented a diameter above 100 µm, a threshold considered as the maximum size for hepatic organoids [26,29]. On the contrary, in Na_2_CO_3_ scaffolds, cells filled up the pores generating thin and elongated cell clusters (Figure 3a middle panel) with a broader range of sizes due to the presence of both large and thin pores generated by CO_2_ release. Median volumes for Na_2_CO_3_-10 and Na_2_CO_3_-15 scaffolds were, respectively, 114.9 and 114.6 µm^3^ (Figure 3b). In the combined scaffolds, distribution profiles of cell cluster volumes were similar to the distribution observed within NaCl scaffolds although volume distributions were slightly more heterogeneous (Figure 3a–c), likely due to the presence of a small number of large and thin pores generated by CO_2_ release. Cell cluster median volumes were 55 µm^3^ for Combined-15 scaffolds, and 56.1 µm^3^ for Combined-20 scaffolds (about 47 µm diameter if considered spherical), but appear less spherical in the gel depth (z-direction) (Figure 3c). We noticed that the cells organized in spherical aggregates in combined scaffolds did not fill completely the volume of pores while they filled up the large elongated pores in Na_2_CO_3_ scaffolds (Figure 3a).

The number of clusters per mm^3^ was also evaluated and correlated with cell cluster volume (Figure 3b) as an index of loading efficiency and cell distribution within the scaffolds. As expected, the number of cell clusters per field of view was inversely proportional to cell cluster volume: a high number of cell clusters was detected within NaCl and Combined scaffolds, while the number was lower in Na_2_CO_3_ scaffolds (Figure 3c). To identify the scaffolds presenting the higher loading efficiency, the metabolic activity of cells in 3D was assessed 24 h after seeding (Figure 3d). This analysis demonstrated a slightly lower metabolic activity in Na_2_CO_3_-15 and Combined-15 than with other formulations, most likely due to, respectively, the presence of too large pores that do not retain cells and too small non-interconnected pores. Taken together, these results showed that two different types of scaffolds were formed, the first one formed with NaCl (or combined formulations) presented small and ovoid pores generating numerous small and spherical cell clusters, and the second type of scaffold with Na_2_CO_3_ exhibited thin and elongated pores producing cell clusters with similar elongated geometries. We therefore chose to compare for the following experiments cell viability and functionality in NaCl and Na_2_CO_3_-10 scaffolds that exhibited the two highest loading efficiencies and a clear difference in terms of cell cluster sizes and shapes. 

### 2.3. Cell Viability 

To assess the viability and proliferation capacity of HepG2 cells organized in small and spherical (NaCl scaffolds) versus large and thin clusters (Na_2_CO_3_-10), we analyzed overtime cell metabolic activity using a resazurin-based assay, and viability using a live–dead assay. To be noted, storage modulus measured by Dynamic Mechanical Analysis at 1Hz in the viscoelastic linear domains were similar on NaCl and Na_2_CO_3_-10 scaffolds, respectively 5010 ±720 Pa and 4390 ± 210 Pa, implying that cell behavior could not be attributed to a difference in the mechanical rigidity of the scaffolds.

**Figure 4:**
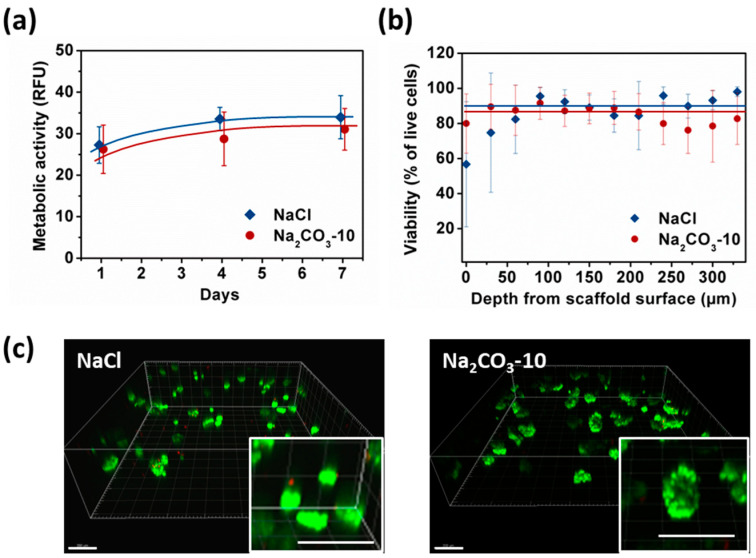
Analysis of HepG2 viability and metabolic activity over seven days within NaCl and Na_2_CO_3_-10 scaffolds. (**a**) Cell metabolic activity of HepG2 within NaCl (blue) and Na_2_CO_3_-10 (red) scaffolds at day one, four and seven. (**b**) Percentage of viable cell within NaCl and Na_2_CO_3_-10 scaffolds as function of distance from the scaffold surface at day seven. Statistics were performed using ANOVA with Tukey’s post-test. No significant difference was observed. (**c**) Representative 3D images of live-dead analysis of NaCl (top panel) and Na_2_CO_3_ (bottom panel) scaffolds at day seven. Live cells were stained with Calcein AM (green) and dead cells are detected with Ethidium homodimer (red). Scale bar: 200 µm.

Metabolic activity was similar at day one for NaCl and Na_2_CO_3_-10 scaffolds (Figure 4a). In NaCl scaffolds, metabolic activity increased significantly from day one to day four and seven reaching a plateau, i.e., HepG2 cells stopped proliferating but remain viable. In Na_2_CO_3_-10 scaffolds, metabolic activity increased slightly but continuously over seven days. Therefore, cell proliferation was limited within both types of scaffolds but cells remain alive for at least seven days. After seven days of culture, no statistical difference was noted between these two scaffold formulations (Figure 4a). We analysed the cell viability using live–dead assay, especially in the gels depth where diffusion could be limited, and within clusters. The overall viability within the 3D hydrogels at day seven was 86.8 ± 6.3% for NaCl scaffold and 90.5 ± 5.9% in Na_2_CO_3_-10 scaffolds. Confocal images demonstrated that the live–dead ratio remained constant in the scaffold depth up to the maximum possible analysis at 300 µm by confocal microscopy (Figure 4b) for both scaffolds. The homogeneous distribution of the cell clusters was evidenced within both scaffolds (Figure 4c). When zooming in on cell clusters, it appears that the visible dead cells were not located in the center of clusters, but they were often individualized isolated cells (Figure 4c, inserts). We hypothesized that individual cells did not survive because they cannot adhere to the scaffolds whereas they can live when forming a cell cluster. Hence, the viability was similar between small and spherical cell clusters (NaCl scaffold) and in elongated and large clusters (Na_2_CO_3_ scaffold), implying a sufficient diffusion of nutrients and oxygen.

### 2.4. Cell Functionality in Porous Scaffolds

**Figure 5:**
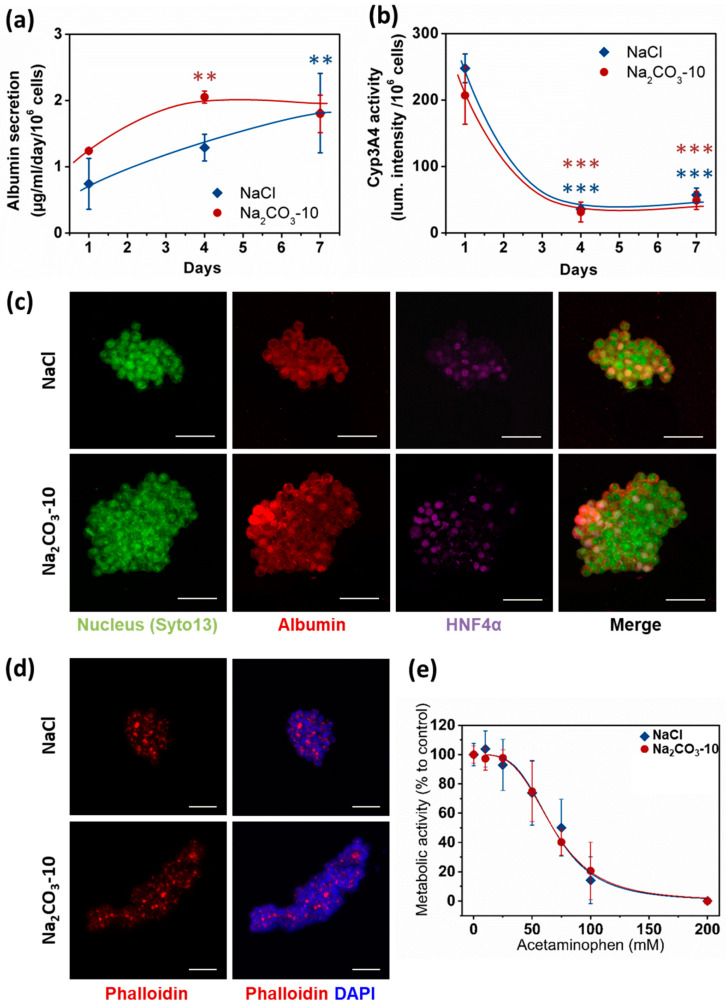
HepG2 cell functionality in 3D within NaCl and Na_2_CO_3_-10 scaffolds. (**a**) Quantification of albumin secretion in the medium for 24 h after one, four and seven days using ELISA. (**b**) Quantification of P450 cytochrome CYP3A4 activity overtime. (**c**) Confocal images (Maximum intensity projection) of typical cell clusters showing the expression of Albumin (red) in the cytoplasm and HNF4α (purple) localized in the nucleus for scaffolds prepared with NaCl (top panel, 30µm total thickness) and Na_2_CO_3_-10 (bottom panel, 80µm total thickness) at day seven. (**d**) Confocal images at day seven (Maximum intensity projection) of typical cell clusters showing the aggregation of Actin (red) in between cells for scaffolds prepared with NaCl (top panel, 49 µm total thickness) and Na_2_CO_3_-10 (bottom panel, 94 µm total thickness). (**e**) Acetaminophen-induced toxicity at day seven on HepG2 cells after 24 h treatment in NaCl and Na_2_CO_3_-10 scaffolds. Scale bars: 50 µm. Statistical analysis was performed using ANOVA with Tukey’s post-test. ** *p* < 0.01, *** *p* < 0.001.

Apart from viability, size and geometry of cell clusters are known to modulate cell differentiation and hence organoid functionality [26,29]. Therefore, albumin secretion, CYP3A4 activity and expression of Albumin and HNF-4α were analyzed as typical hepatic function markers. We quantified albumin secretion in cell culture supernatants after one, four and seven days. At day one, no statistical difference was observed between the two types of scaffolds, although the albumin quantity was slightly higher in Na_2_CO_3_-10 scaffold than NaCl scaffold (respectively 1.24 ± 0.02 and 0.74 ± 0.38 µg/mL/10^6^ cells). Albumin production increased overtime in both scaffolds to reach similar values at 1.80 ± 0.28 µg/mL/10^6^ cells in Na_2_CO_3_-10 scaffolds and 1.81 ± 0.6 µg/mL/10^6^ cells in NaCl scaffolds at day seven (Figure 5a). These albumin concentrations are in agreement with several others studies using HepG2 cells [48,49,50]. CYP3A4 activity (Figure 5b) exhibited a profile inversely proportional to albumin secretion, also in agreement with the literature [51,52,53]. In line with these data, we also observed cytoplasmic expression of albumin in HepG2 cells within both types of scaffolds, and nuclear expression of HNF-4α in most cells at day seven (Figure 5c). Moreover, phalloidin staining revealed actin aggregates bounded by HepG2 cells (Figure 5d), a phenomenon coinciding with the development of bile canaliculi [54,55,56,57]. To further confirm that these cell clusters were functional, we performed a dose–response analysis of acetaminophen toxicity in the 3D constructs (Figure 5e). In both NaCl and Na_2_CO_3_-10 scaffolds, HepG2 cells were sensitive to increasing concentrations of acetaminophen with EC_50_ of 67.70 and 67.75 mM, respectively. As acetaminophen is catalyzed by CYP3A4 to a toxic intermediate, this suggests that 3D constructs in both scaffolds have an active and functional CYP3A4 and were able to detect liver toxic compounds. Overall, these data demonstrate that cell clusters formed in 3D scaffolds present the main characteristics required for hepatic constructs.

## 3. Discussion

The main goal of this study was to analyze the effect of sizes and geometries of hepatic constructs on cell functionality using porous scaffolds. Scaffolds were prepared by cross-linking of polysaccharides in the presence of different porogen agents (NaCl or Na_2_CO_3_) to obtain various microstructures of different sizes and geometries. These scaffolds made of polysaccharides are easy to produce and to handle. They are transparent, allowing direct microscopic observation, and they can be stored at room temperature long term before use. We used a patented technique based on the combination of porogen addition and freeze drying process [41,42]. Concentrated solutions of high molecular weight polysaccharide solutions being very viscous; a maximum of 20% (*w/w*) of porogen could be added to the polysaccharide solution (Table 1). Scaffolds formed with NaCl presented small and ovoid pores originating from ice crystal formation during the freeze drying process [58,59]. In both Na_2_CO_3_ and Combined scaffolds (NaCl+Na_2_CO_3_), two types of porosity were observed: large and very thin pores coming from the release of CO_2_ after crosslinking [60,61] and smaller pores arising from the freeze-drying step. The small pores displayed in Na_2_CO_3_ were not visible after hydration, most likely due to polymer swelling in water. Indeed, these scaffolds absorb more than 10 times their weight in water (Table 1), but their total volume decrease by a factor of about 2.8 times. This suggests a high swelling of polysaccharide chains thereby concealing the small pores. In Combined-20 scaffolds compared to Na_2_CO_3_-10 scaffolds, which both contain the same amount of Na_2_CO_3_, we observed a higher number of small pores and a lower number of thin elongated pores. Hence, the presence of NaCl in the initial solution affect pore formation in Na_2_CO_3_ scaffolds.

The percentage of porosity between 30% and 50% and pore geometry of all scaffolds appear adapted for cell loading and infiltration. Besides, recent results also suggest that there is also nanoporosity that will allow nutrients and oxygen diffusion [59] which is not observable by confocal microscopy because of its technical limitations. The increase in porogen amount increases slightly but not significantly the total porosity of scaffolds. Upon immersion in PBS, the polysaccharide chains swelling in the center of the scaffolds fill in the pores. Therefore, the increase in pore size due to the increase in porogen amount is partially masked by the swelling of polymer chains. Phosphorous content analysis showed that phosphorous amount was higher in NaCl scaffolds than in all other formulations. Even though phosphorous is not strictly instructive on the effective bounds between two polysaccharide chains, i.e., crosslinking density, previous studies have shown that the mesh size calculated from phosphorous amount correlate with rheological behavior [46,59] and therefore can be used to appraise crosslink ratio. Although STMP crosslinking reaction is still not fully understood in such a complex system, we know that the STMP-mediated formation of phosphorous bound between hydroxyl groups in alkaline solution brings into play different mechanisms. Polysaccharides diluted in alkaline solution result in immediate alcoholate formation that can attack and open STMP resulting in polysaccharide-bound sodium tripolyphosphate. Next, another alcoholate can attack polysaccharide-bound sodium tripolyphosphate creating a phosphoester bond between two saccharide units. However, alcoholate attacks on phosphorous links are in competition with NaOH, suggesting that crosslinking reaction is very sensitive to pH [62,63]. We hypothesized that the presence of Na_2_CO_3_ in the starting polysaccharide solution, modulates osmotic pressure and therefore affects crosslinking reactions. This would explain the significantly higher phosphorous content observed for NaCl scaffolds compared to all other formulations. Swelling ratio and degradation time was increased when increasing porogen amount, especially for Combined-20 scaffolds. The biphasic in vitro degradation pattern of these scaffolds demonstrates that different factors govern enzymatic degradation rates. Firstly, the scaffold porosity is a major factor determining degradation rate because of enzyme diffusion and provided surface area. The lower porosity of NaCl scaffolds can explain the slow degradation compared to all others scaffolds. However, Na_2_CO_3_ and Combined scaffolds present a similar porosity but different degradation times. Although the surface area was not quantified in this study, the presence of small pores in Combined scaffolds compared to Na_2_CO_3_ scaffolds likely increases surface area and therefore degradation rate. A second parameter is the availability of enzyme-binding motifs in the polysaccharide chains, influenced particularly by ionic interactions such as phosphate groups. Consequently, the less porous and more crosslinked scaffolds (NaCl) present a slower degradation rate, whereas the more porous scaffolds, presenting a large surface area, are degraded faster. 

When HepG2 cells were seeded, we observed that cells were viable within all formulations of polysaccharide scaffolds forming various cell clusters geometries. The biophysical characteristics of 3D scaffolds highly influence cell behavior in terms of adhesion, viability, proliferation and functionality [64,65]. In the particular case of organoids in hydrogels that do not promote cell adhesion, cells spontaneously self-organize and therefore porosity—which controls the number of cells in a confined space—is an essential characteristic that dictates cell behavior [38,66]. Cell loading/infiltration is also the crucial initial step that will influence the performance of a 3D scaffold to support organoid development. In this study, dry scaffolds could be easily impregnated with a cell suspension, thanks to the hygroscopic properties of polysaccharides and pore interconnectivity. Moreover, cells were uniformly distributed, which is essential for future applications in tissue engineering. A higher cell loading was observed in NaCl scaffolds which correlated with small pores and a high swelling ratio. Besides, the large pores and more open surfaces observed in Na_2_CO_3_ or Combined scaffolds can explain the lower cell retention in the scaffolds and the variability of cell clusters sizes. HepG2 cells remain viable equally in all scaffolds for more than a week. Considering a doubling time of 48 h for this cell line on usual 2D substrates, proliferation is moderate in the 3D scaffolds, probably owing to an efficient cell loading and lack of available space but also because cells do not adhere to the scaffold surface. Moreover, formation of cell spheroids might reduce proliferation, since cells engage mostly in matrix production [66]. Therefore, the limited proliferation is not detrimental when considering the application of these scaffolds for organoid production with different cell types such as primary hepatocytes or differentiated hepatocytes from iPS which do not proliferate. 

After seven days in culture, we demonstrated that cell clusters sizes match pore size and shape. We observed a noticeable difference of cell clusters size between the scaffolds produced using Na_2_CO_3_ where cell clusters are large compared to the other formulations displaying a majority of smaller clusters. This observation correlates with the presence of small pores in combined scaffolds. In contrast, increasing porogen amount did not result in a significant change of scaffold porosity nor cell clusters sizes. In organoids, cells usually experience mass transfer limitations because of the absence of a vascular network [6,11,20]. It was previously demonstrated that primary hepatocytes viability exponentially decreased at the center of spheroids of about 100–150 µm to the spheroid surface due a decrease in oxygen [26,67,68,69]. In our study, more than 80% of hepatic constructs present a size lower than 100 µm in diameter if considering a spherical shape. Moreover, since the largest constructs present ovoid shapes, the volume is not a limitation to cell viability, as the maximum distance of cells located in the center of organoids are only a few cells away from the medium. Interestingly, viability was not impaired when HepG2 cells fill up pores completely. This possibly relates to the appropriate diffusion of oxygen and nutrients within the polysaccharide network. Indeed, diffusion coefficients of oxygen in hydrogels are dependent on the polymer concentration/density but remain in the same order of magnitude than diffusion in water about 10^−9^ m^2^/s [70,71,72]. Larger molecules such as glucose or albumin exhibit lower diffusivity with coefficients expected to be about 10^−10^ to 10^−11^ m^2^/s if we consider that cell consumption is negligible [70]. Therefore, all types of scaffolds seem adapted for the development of viable small 3D cell constructs. Moreover, dynamic mechanical analysis revealed that scaffold elasticity is in the order of magnitude of elasticity of physiologic livers (4 kPa) [73,74,75,76]. 

HepG2 constructs in NaCl and Na_2_CO_3_ scaffolds differentiate and show specific functions of hepatocytes, such as expression of HNF4α, production of albumin and CYP3A4 activity. Hepatic constructs from both scaffolds demonstrate a profile of albumin secretion inversely proportional to CYP34A, in agreement with data from the literature [51,52]. We found a low CYP3A4 activity of HepG2 constructs in our scaffolds similar to the limited activity observed for small size cell spheroids of less than 200 μm [66]. For larger HepG2 spheroids, hypoxic areas in HepG2 3D constructs increases in the core and subsequently increases CYP3A4 activity [53]. In our study, no hypoxia or cell death was observed in 3D constructs which could explain the reduced activity of CYP3A4 overtime. Moreover, an acetaminophen-induced cytotoxicity assay demonstrated that HepG2 cells are sensitive in a concentration-dependent fashion. CYP3A4 activities are responsible of 30% to 50% metabolism of medications by the P450 system in humans [77], including acetaminophen, the most common used analgesic antipyretic agent and the major cause of toxic liver injury in Western countries. In addition, the presence of actin condensation between HepG2 cells suggest that the cells polarize to form bile canaliculi [54,55,56,57]. Although some of the structures observed appear as vacuoles, some canaliculi are connected therefore indicating cell clusters organization and early signs of polarization. 

Therefore, our data suggest that hepatic constructs formed in both NaCl and Na_2_CO_3_ scaffolds are viable and functional. As polysaccharide hydrogels are easily adjustable in terms of microstructures and surface properties [40,46,47], these scaffolds are very promising for drug-toxicity studies but also for liver regenerative medicine as they can be GMP produced. Moreover, this platform can be adapted for other types of organoids with single or multiple cell types. This study is a first proof a concept for the use of these scaffolds in cell-self-assembly. Future works will be needed with more suitable cell types and to confirm these results in vitro and in vivo for hepatic organoid production and long-term viability and functionality.

## 4. Materials and Methods

### 4.1. Preparation of Scaffolds

Polysaccharide-based scaffolds were prepared as described in [41,42]. Briefly, a mixture of pullulan (Hayashibara Co., Ltd., Okayama japan; Mw 200 kDa) and dextran (Pharmacosmos, Holbaeck, Denmark; Mw 500 kDa) with a ratio 75:25 was prepared in water. Sodium chloride (NaCl) and/or sodium carbonate (Na_2_CO_3_) were added to the polysaccharide solution (Table 1). The polysaccharide blend was crosslinked under alkaline conditions (1 M sodium hydroxide) using 3% (*w/v*) STMP (Sigma) and casted between two glass plates before incubation at 50 °C for 20 min. The resulting gels were punched in wet state to obtain 5mm diameter and 1mm thick scaffolds that were equilibrated to neutral pH using 10× PBS and washed extensively with distilled water for at least 24 h to remove the porogen agent and the excess of salts. When Na_2_CO_3_ was used, scaffolds were firstly transferred into 20% acetic acid to allow CO_2_ gas release before neutralization and rinsing. All scaffolds were freeze-dried in water or NaCl 0.025% (*w/v*) for the scaffolds with NaCl and stored at room temperature until use. The solubility and scaffold formation was evaluated with increasing quantities of porogens. For porosity analysis, 1% fluorescein isothiocyanate (FITC)-labeled dextran 500 kDa (TdB Consultancy, Uppsala, Sweden) was added to the polymer solution before crosslinking. 

### 4.2. Characterization of Biophysical Properties of Scaffolds

#### 4.2.1. Phosphorous Content Analysis

About 20 mg of each scaffold were degraded with 1 mL of 10% nitric acid and incubated at 105 °C for 3 h. Subsequently, 0.4 mL of 14.7 M nitric acid, 2 mL of 10 mM ammonium metavanadate, and 2 mL of 40 μM ammonium molybdate tetrahydrate were added into each sample. Phosphorous ions concentration was then determined by spectrophotometry at 405 nm using a phosphoric acid standard curve. Samples were run in triplicates and results were expressed as mean values ± SD.

#### 4.2.2. Swelling Behavior of the Scaffolds

Scaffolds were weighed before (w_i_) and after (w_f_) impregnation in 1X PBS for three days. Swelling ratio was determined by the following equation: Sw = (w_f_ − w_i_)/w_i_

#### 4.2.3. In Vitro Enzymatic Degradation

Having determined scaffolds initial weight in 1× PBS, scaffolds were incubated at 37 °C in a solution of pullulanase (Sigma, E2412) and dextranase (Sigma, D0443) diluted, respectively, 1.5/10 and 0.75/10 in 1× PBS. Every 5 min scaffolds were retrieved and weighed after removing the excess of liquid using filter papers. Scaffolds degradation was followed overtime until 100% mass loss. Samples were run in triplicate and results were expressed as mean values ± SD.

#### 4.2.4. Dynamic Mechanical Testing

Hydrogels of 25 mm in diameter and 1 cm height were prepared and hydrated in PBS for two weeks before analysis on Discovery HR2 (TA Instruments, Guyancourt, France) rheometer. Shear stress measurements were performed in PBS under oscillation mode using 25 mm aluminum plate geometries fitted with sandpaper, grit size 600, to avoid sample slippage during measurement. Viscoelastic linear domains were determined by an amplitude sweep between 0.01% to 10% strain with a constant frequency of 1 Hz at 25 °C. To ensure minimal compression, normal force was kept constant at 0.3 +/− 0.1 N. Samples were run in triplicate and results expressed as mean values ± SD.

### 4.3. Characterization of Scaffolds Microstructure

The microstructure of the freeze-dried scaffolds was analyzed using Scanning Electron Microscopy (SEM). Scaffolds were cut vertically using a razor blade and glued to sample holders to observe the inner structure in a SEM JSM-IT100 InTouchScope™ (Jeol, Croissy, France)under low vacuum mode (40 Pa) at a 20-kV acceleration voltage.

For porosity analysis in wet state, scaffolds containing FITC-Dextran were hydrated in 1× PBS and analyzed using 2-photon Leica SP8 microscope fitted with a HCX IRAPO L 25X objective (CRI—U1149 Imaging facility). Confocal slices (3 µm deep) were imaged over about 150–200 µm in the scaffold depth with biphoton excitation at 750 nm. A macro was written specifically to determine polymer and pore volumes in 3D Stacks using FIJI freeware. Briefly, the images intensities were normalized in the gel depth before applying an intensity threshold to obtain a binary image. The plugging 3D object counter [78] was used to detect objects corresponding to the pores in the scaffolds and the total volume and pores volumes were calculated and compiled. Total volume analyzed per scaffold was at least 0.15 mm^3^, with at least six scaffolds analyzed for each condition.

### 4.4. Cell Culture and Loading into 3D Scaffolds

HepG2 cells (Human hepatocellular carcinoma) were obtained from ATCC^®^ (HB8065™) and cultured in Dulbecco’s modified Eagle’s Medium DMEM 4.5 g/L glucose supplemented with 10% Fetal Bovine Serum and 1% antibiotic-antimycotic (Sigma, France). The scaffolds were sterilized under UV light for 30 min before cell culture. Cell loading into scaffolds was performed using syringe-induced vacuum, adapted from [79]. Briefly, all scaffolds were introduced in a syringe along with a cell suspension; the plunger was introduced and the syringe tip was closed using a 3-way valve. Vacuum was induced by moving the plunger of about 3 cm up and down until the scaffold were fully impregnated, i.e., when they become transparent. Maximum cell loading efficiency was determined beforehand and a seeding density of approximately 100,000 cells per scaffold, i.e., 1428 cells/µL, was chosen. Scaffolds were placed in 24-well plates with 1 mL DMEM.

### 4.5. Cell Clusters Size and Shape Analysis

Cellularized scaffolds were fixed with paraformaldehyde 4% for 1 h at room temperature, rinsed three times with 1× PBS, permeabilized with 0.1% Triton X100 for 1h followed by incubation in a solution of Phalloidin (Phalloidin-TRITC 1/200, Sigma) and DAPI (Sigma, 1 µg/mL) for 1 h at room temperature. 3D stacks were obtained using an inverted Zeiss LSM 780 confocal microscope fitted with a 10× objective (CRI—U1149 Imaging facility) over up to 400 µm in the scaffold depth to analyze cell clusters size and shape. Images were reconstructed in 3D using Imaris software and a semi-automatic analysis allowed the detection of objects corresponding to the cell clusters. A background intensity threshold and minimal exclusion size of about one cell were set manually, individual items too close to be separated automatically were split manually and filters were applied to remove objects not entirely in the field of view. The volumes of cell clusters were then evaluated automatically for at least three samples per condition and a total of more than 150 cell clusters were analyzed.

### 4.6. Cell Viability Assay

#### 4.6.1. Live–Dead Analysis

Live–dead analysis was performed at day one, four and seven to analyze the viability in the depth of the scaffolds. Calcein AM (Invitrogen, Les Ulis France) and Ethidium homodimer (Molecular Probes™), were diluted in culture medium at 1/500 and 1/200 ratios, respectively, and incubated with the cellularized scaffolds for 1 h. After rinsing twice with 1× PBS, imaging was performed using Zeiss LSM 780 confocal microscope (CRI—U1149 Imaging facility) over about 300 µm in the depth of the scaffold. The percentage of live and dead cells was determined in the gel depth. Briefly, cells were counted manually on 3 µm deep confocal slices every 30 µm on at least five different samples for each condition.

#### 4.6.2. Resazurin Assay

Metabolic activity quantification was performed at day one, four and seven using the In Vitro Toxicology Assay Kit (MTT based, Sigma-Aldrich France). Seeded scaffolds were transferred to a 48-well plate and incubated with 0.5 mL of resazurin for 2.5 h at 37 °C. Fluorescence of the resulting solution was measured Using Infinite M200 Pro, TECAN^®^ plate reader (560Ex/590Em). All samples were run in triplicate in triplicate in three different experiments.

### 4.7. Cell Functionality Analysis

#### 4.7.1. Albumin Secretion

Albumin secretion in cell culture medium was determined overtime by an ELISA assay (Thermo Scientific™) according to the manufacturer’s recommendations. Standards and samples were incubated in the Anti-Human Albumin Precoated 96-well Strip Plate for 2.5 h at room temperature and washed four times before the addition of a biotinylated antibody. After rinsing, the streptavidin-HRP reagent was incubated. Finally, TMB substrate was added and the absorbance was measured at 450 and 550 nm using the Infinite M200 Pro, TECAN^®^ plate reader (Tecan, Männedorf, Switzerland). All samples were run in triplicate.

#### 4.7.2. CYP3A4 Activity

Cytochrome P450 3A4 (CYP3A4) activity was analyzed using P450-Glo™ CYP3A4 Assay (Promega). Briefly, a pro-luciferin substrate was added to the cell culture medium and incubated for 1 h at 37 °C. Aliquots of supernatants were collected and incubated with the luminogenic detection reagent before luminescence reading using TECAN^®^ plate reader. All samples were run in triplicate.

#### 4.7.3. Cell Number Assessment

To normalize cell activity to number of cells, total protein content was determined using Micro BCA™ Protein Assay Kit (Thermo Scientific™). Hydrogels were digested using pullulanase and dextranase diluted in 1X PBS (respectively 1/10 and 1/20) for about 40 min at 37 °C. Cells were rinsed twice with 1X PBS and lysed in 350 µl of cold TRIS-EDTA lysis buffer (Invitrogen™ T11493) and homogenized using 25-G needles. Cell numbers were calculated thanks to a standard curve of known cells concentrations and used to normalize albumin production and CYP3A4 activity.

#### 4.7.4. Albumin and HNF4α Staining

Scaffolds containing HepG2 cell clusters were fixed after seven days in paraformaldehyde 4% and rinsed before blocking and permeabilization in 1% BSA 0,1% Triton for 3 h at room temperature. Anti-albumin primary antibody (Tebu-bio Cedarlane, 007CL2513A, 1/200) was incubated for 24 h at 4 °C in blocking solution, followed by extensive rinsing. Anti-NHF4α antibody (Ozyme Atlas, HPA004712, 1/150) was then incubated for 3 h at room temperature and finally after rinsing the secondary antibodies, AlexaFluor 555 anti-mouse Alexa Fluor 647 anti-rabbit (Invitrogen™, 1/200) were applied for 1.5 h at room temperature. Samples were observed using Zeiss LSM 780 confocal microscope fitted with a 10× objective (CRI—U1149 Imaging facility).

### 4.8. Acetaminophen Dose-Toxicity Analysis

Acetaminophen dose response was analyzed in the 3D scaffolds at day seven. HepG2 cells were treated for 24 h with increasing concentrations of acetaminophen (Sigma). As acetaminophen is not fully solubilized above 100 mM, 200 mM acetaminophen solutions were prepared separately for each well for reproducibility purposes. Cell viability was quantified using resazurin-based metabolic activity (2 h incubation at 37 °C). Fluorescence was measured using Infinite M200 Pro, TECAN^®^ plate reader (560Ex/590Em). Data were expressed as a percentage of unstimulated cells viability. Fits of cell viability concatenated data were drawn using Origin software to determine EC_50_ (half maximal effective concentration). Experiments were done in triplicate with 8–16 samples per experiment.

### 4.9. Statistical Analysis

All experiments were carried out at least in triplicate and statistical analysis was performed using Student’s *t*-test for all analysis except for viability and functionality analysis overtime where ANOVA with Tukey’s post-test was used. Statistical significance was indicated as * *p* < 0.05, ** *p* < 0.01, *** *p* < 0.001.

## 5. Conclusions

The aim of this project was to produce viable and functional hepatic constructs within polysaccharide-based scaffolds with different structures, and to determine whether these structures could affect cell morphology, viability and functionality. Pullulan-dextran hydrogels devoid of any components of human or animal origin were prepared easily in the absence of organic solvent using pharmaceutical-grade polysaccharides and a process that can be translated in industry to produce GMP scaffolds. Our results showed that different porosities can be created depending on the porogen. Cell loading efficiency was inversely proportional to porosity and hepatocytes formed constructs that match with pore size and geometry. In small spherical pores, cells mostly condensed to form spherical cell clusters, whereas in large elongated pores, cells formed long and thin cell constructs. In all types of scaffolds, HepG2 cells were highly viable, proliferate for seven days and present characteristics of functional organoids such as albumin production, CYP3A4 activity and sensitivity to acetaminophen. Therefore, hepatic organoids in such a 3D microenvironment with adjustable properties can be adapted for in vitro drug screening, in vivo implantations and extracorporeal devices.

## Figures and Tables

**Table 1:**
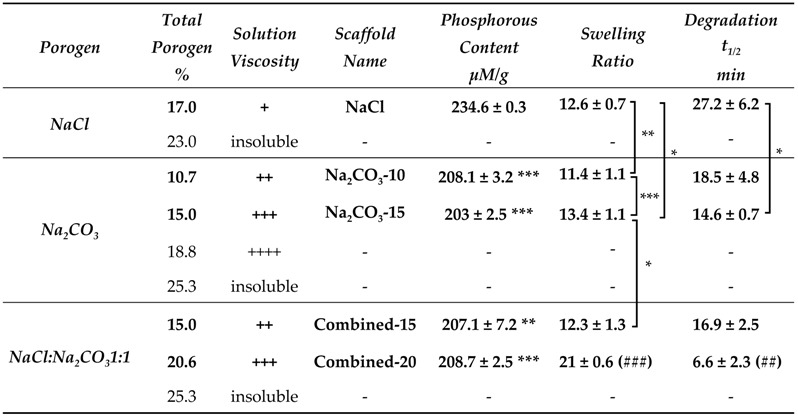
Effect of polysaccharide formulations (porogen type and weight amounts) on scaffold production, phosphorous content (*n* = 3), swelling ratio (*n* > 6) and in vitro enzymatic degradation (t_1/2_, time to degrade 50% of the scaffold weight). Statistical analysis using student *t*-test. * *p* < 0.05, ** *p* < 0.01, *** *p* < 0.001 denote statistical significance against NaCl scaffold. ^##^ *p* < 0.01, ^###^ *p* < 0.001 denote statistical significance against all other scaffolds.

## Abbreviations

NaCl	Sodium Chloride
Na_2_CO_3_	Sodium carbonate
PBS	Phosphate Buffer Saline
SEM	Scanning Electron Microscopy
STMP	Sodium Trimetaphosphate
CYP3A4	Cytochrome P450 3A4
EC_50_	Half Maximal Effective Concentration
